# Genotyping coronaviruses associated with feline infectious peritonitis

**DOI:** 10.1099/vir.0.000084

**Published:** 2015-06

**Authors:** Catherine S. Lewis, Emily Porter, David Matthews, Anja Kipar, Séverine Tasker, Christopher R. Helps, Stuart G. Siddell

**Affiliations:** ^1^​ School of Cellular and Molecular Medicine, University of Bristol, Bristol BS8 1TD, UK; ^2^​ School of Veterinary Sciences, University of Bristol, Langford, Bristol BS40 5DU, UK; ^3^​ Institute of Veterinary Pathology, Vetsuisse Faculty, University of Zurich, Winterthurer Strasse 268, 8057 Zurich, Switzerland

## Abstract

Feline coronavirus (FCoV) infections are endemic among cats worldwide. The majority of infections are asymptomatic or result in only mild enteric disease. However, approximately 5 % of cases develop feline infectious peritonitis (FIP), a systemic disease that is a frequent cause of death in young cats. In this study, we report the complete coding genome sequences of six FCoVs: three from faecal samples from healthy cats and three from tissue lesion samples from cats with confirmed FIP. The six samples were obtained over a period of 8 weeks at a single-site cat rescue and rehoming centre in the UK. We found amino acid differences located at 44 positions across an alignment of the six virus translatomes and, at 21 of these positions, the differences fully or partially discriminated between the genomes derived from the faecal samples and the genomes derived from the tissue lesion samples. In this study, two amino acid differences fully discriminated the two classes of genomes: these were both located in the S2 domain of the virus surface glycoprotein gene. We also identified deletions in the 3c protein ORF of genomes from two of the FIP samples. Our results support previous studies that implicate S protein mutations in the pathogenesis of FIP.

## Introduction

**Fig. 1.**
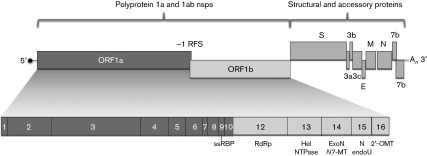
Genomic organization of FCoV. Genomic ORFs are shown as boxes. Only pp1ab is shown as a translation product of the genomic RNA. The non-structural proteins nsp1–11 are translated from ORF1a (dark grey shading) and translation of the ORF1b proteins (nsp12–16) occurs following −1 ribosomal frameshifting (RFS). The nsp11 protein is not depicted as it represents a short (9 aa) carboxyl extension of nsp10. nsp9, ssRNA-binding protein (ssRBP); nsp12, RNA-dependent RNA polymerase (RdRp); nsp13, helicase (Hel) and NTPase; nsp14, 3′→5′ exoribonuclease (ExoN) and *N*7-methyltransferase (*N7*-MT); nsp15, uridylate-specific endonuclease (NendoU); nsp16, 2′-*O*-methyltransferase (2′-*O*MT).

Coronaviruses are enveloped, positive-sense RNA viruses. They are generally responsible for mild enteric and respiratory infections, but they can also be associated with severe disease in both humans and animals (Masters & Perlman, 2013). Coronaviruses are now recognized as emerging viruses with a propensity to cross into new host species, as has been shown by the recent outbreaks of severe acute respiratory syndrome and Middle East respiratory syndrome (Coleman & Frieman, 2014). As illustrated in Fig. 1 for feline coronavirus (FCoV), two-thirds of the coronavirus genome encodes proteins involved in viral RNA synthesis. The majority of these proteins are encoded in two 5′-proximal, overlapping ORFs, ORF1a and ORF1b, and are translated as the polyproteins pp1a and pp1ab, which are then processed by virus-encoded proteinases into 16 non-structural proteins (Ziebuhr, 2005). The remainder of the genome encodes the virus structural proteins (S, E, M and N), as well as accessory proteins that are not essential for replication in cell culture. The structural and accessory proteins are translated from a 3′ co-terminal nested set of subgenomic mRNAs (Perlman & Netland, 2009).

The coronavirus surface or spike (S) glycoprotein is a typical class 1 viral fusion protein and has a central role in the biology of coronavirus infection. Structurally, the protein can be divided in to an amino-proximal half, the S1 domain, which contains the receptor-binding domain, and a carboxyl-proximal half, the S2 domain, which contains elements involved in membrane fusion. These elements include heptad repeats, a fusion peptide and a carboxyl-terminal, hydrophobic transmembrane domain (Heald-Sargent & Gallagher, 2012). In many coronaviruses, the S1 and S2 domains are cleaved from each other by a cellular, furin-like enzyme (de Haan *et al.*, 2004). The S protein is also the location of both B- and T-cell epitopes that are important in virus neutralization and the recognition of virus-infected cells (Reguera *et al.*, 2012; Satoh *et al.*, 2011).

FCoVs form two antigenically distinct serotypes: serotype 1, which is difficult to propagate in cell culture, and serotype 2, which is the consequence of a double recombination between type 1 FCoV and canine coronavirus (Herrewegh *et al.*, 1998) and is relatively easy to propagate in cell culture. FCoV infections are endemic among cats worldwide, and serological and molecular studies confirm that serotype 1 FCoVs predominate (Pedersen, 2014b). In the UK, about 40 % of domestic cats have been infected with FCoV, and in multi-cat households, this figure increases to almost 90 % (Addie, 2000; Addie & Jarrett, 1992). The majority of FCoV infections are asymptomatic or result in only mild enteric disease. However, approximately 5 % of infected cats develop feline infectious peritonitis (FIP), a systemic inflammatory disease that is a frequent cause of death in young cats (Kipar & Meli, 2014). Currently, there is no protective vaccine or effective treatment for FIP (Pedersen, 2009, 2014a).

The most important questions in FCoV research are why some infected animals remain relatively healthy whilst others develop FIP, and what the role of the virus is in the development of disease. It is now widely accepted that, in the vast majority of cases, cats are infected by the faecal–oral route with avirulent FCoV strains circulating in the cat population. Initially, this virus replicates predominantly in the intestinal epithelium and is shed with the faeces. Nonetheless, it often leads to systemic infection via monocyte-associated viraemia (Kipar *et al.*, 2010; Meli *et al.*, 2004; Porter *et al.*, 2014). At this stage, however, the systemic infection is characterized by a relatively low level of virus replication and infection can be maintained for a prolonged period of time, possibly involving recurrent viraemic events, without apparent disease (Kipar *et al.*, 2010). During replication in the intestine or, potentially, within monocytes/macrophages (Pedersen *et al.*, 2012), the virus undergoes mutation, and viruses with an enhanced tropism for monocytes and macrophages emerge. The altered tropism of these mutants results in their ability to maintain effective and sustainable replication in monocytes (Dewerchin *et al.*, 2005). As a direct or indirect result of a higher level of virus replication, this now apparently virulent virus leads to activation of monocytes (Regan *et al.*, 2009), which can then interact with endothelial cells. This, in turn, mediates granulomatous phlebitis and periphlebitis, the morphological hallmark and initiating lesion of FIP (Kipar *et al.*, 2005).

In addition to the virus, the susceptibility of the individual infected cat to disease also plays a significant role, and it has been shown that age, breed, gender, reproductive status and immune response influence the development of FIP (Pedersen, 2014b; Pedersen *et al.*, 2014). For example, the efficacy of early T-cell responses critically determines the disease outcome in cats that have been infected experimentally with a virulent serotype 2 strain, FIPV 79-1146 (de Groot-Mijnes *et al.*, 2005). Furthermore, there is individual variation in the susceptibility of a cat’s monocytes to FCoV (Dewerchin *et al.*, 2005). Also, recently, single-nucleotide polymorphisms in the feline IFN-γ gene have been linked to both resistance and susceptibility to the development of FIP (Hsieh & Chueh, 2014). Clearly, unravelling the relationship between FCoV genotypes and phenotypes and the complex interactions between the virus and host during the development of FIP remains a major challenge.

One facet of this challenge is to determine the mutations that alter the tropism and virulence of FCoV. As a first step, this can be done by comparing the genomic sequences of viruses shed in the faeces of healthy animals and viruses that predominate within tissue lesions of cats that have been diagnosed with FIP. This approach assumes that the most highly abundant genome in a population is responsible for a particular disease phenotype, which is consistent with our current understanding of FIP epidemiology. Using this approach, a recent study by Chang *et al.* (2012) provided evidence for an association between FCoV virulence and amino acid substitutions within the putative fusion peptide of the FCoV S protein. A more detailed examination of samples from FCoV-infected cats that did not have histopathological evidence of FIP led Porter *et al.* (2014) to conclude that these substitutions were indicative of systemic spread, rather than a virus that, without further mutation, is able to cause FIP. As the S protein fusion peptide is involved in the fusion of viral and cellular membranes during virus entry, it seems plausible that changes within this region may be linked to the tropism of the virus.

Similarly, Licitra *et al.* (2013) were able to distinguish between FCoVs in cats with and without FIP on the basis of one or more substitutions in the amino acid sequence that comprises the furin cleavage site within the FCoV S protein. The authors demonstrated that these substitutions modulated furin cleavage and suggested that a possible consequence of the identified substitutions was an enhanced cleavability by alternative, monocyte/macrophage-specific proteases.

Finally, there have been many reports over the years of point mutations and indels in the accessory protein genes of FCoVs and claims that these may be linked to the development of FIP. Prominent among these are reports that truncating and non-truncating mutations in the ORF3c gene occur in a significant proportion of but not all FCoVs associated with FIP (Chang *et al.*, 2010; Pedersen *et al.*, 2012). However, the role of the FCoV 3c protein and any relationship to the development of FIP is still unclear. One view is that functional 3c protein expression is essential for replication in the gut but is dispensable for systemic replication. Thus, once the virus has left the gut, there is no further selection pressure to maintain an intact 3c gene and mutations will accumulate over time. This interpretation does not exclude the possibility that loss or alteration of the 3c protein may enhance the fitness of the virus in the monocyte/macrophage environment, but this is not yet supported by any convincing evidence. Similarly, whilst the genes encoding the 3a, 3b, 7a and 7b proteins clearly have important functions that will impact on virus fitness (Haijema *et al.*, 2004), there is, as yet, no evidence that links specific mutations in these genes to the development of FIP.

In this study, we report the genome sequences of six FCoVs: three from faecal samples from healthy cats and three from tissue lesion samples from cats with confirmed FIP. The six samples were obtained from cats that were resident at a single-site cat rescue and rehoming centre in the UK. Our results support and extend previous studies that implicate S protein mutations in the pathogenesis of FIP.

## Results

### FCoV RNA in faecal and tissue lesion samples

**Fig. 2.**
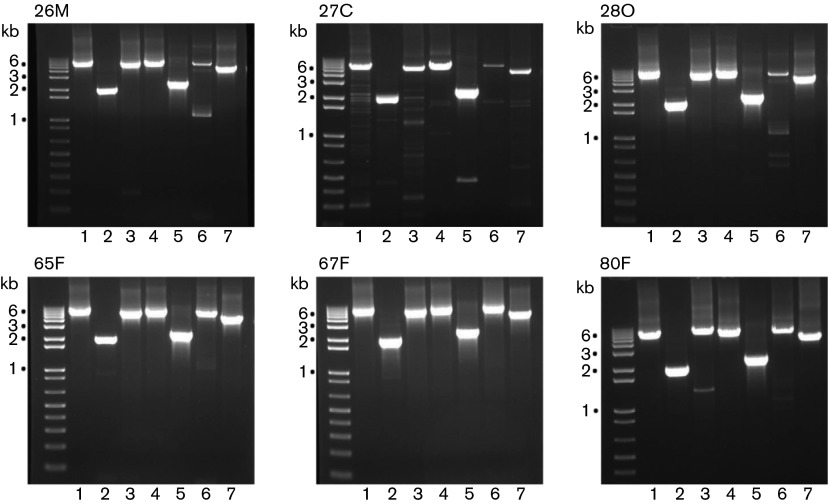
Agarose gel electrophoresis of PCR amplicons 1–7 (lanes 1–7, respectively) for tissue lesion samples 26M, 27C and 28O and faecal samples 65F, 67F and 80F. The 1 kbp DNA plus Ladder (Invitrogen) was used as molecular size markers and the 1, 2, 3 and 6 kbp fragments are indicated.

As a first step, we amplified the FCoV RNA in faecal and tissue lesion samples. The seven amplicons for each of the faecal-derived RNA samples were of the expected size and were produced in approximately equal amounts. In comparison, there was greater heterogeneity in the amplicons obtained from RNA isolated from the FIP tissue lesions (Fig. 2). Specifically, there was more evidence of non-specific products and, especially in the case of amplicon 6, which encompasses the region of the genome encoding the S protein gene, there was less product than expected. In this context, we noted that the cycle threshold (*C*_t_) values were generally higher (i.e. less viral RNA) for faecal samples than for samples from the FIP tissue lesions. The mean *C*_t_ values for the 65F, 67F and 80F faecal total RNA samples were 20.9, 16.9 and 29.0, respectively, and for the 26M, 27C and 28O tissue lesion samples were 14.0, 21.5 and 15.0, respectively. One explanation for the difference in homogeneity of amplicons derived from faecal and lesional samples may be that the samples derived from lesions contained significantly greater amounts of FCoV subgenomic mRNA than the faecal samples, which would be expected to contain mainly virion particles. Also, immunohistochemistry identified a large number of macrophages with abundant viral antigen (i.e. N protein) within the lesions (data not shown). It is therefore very likely that the RNA extracted from the lesions contained much more viral mRNA than the faeces. Thus, in the reverse transcription (RT)-PCRs that involved RNA from tissues, many of the oligonucleotide primers would bind to multiple templates, resulting in a more complex amplicon pattern.

### Assembly of genome sequences

**Fig. 3.**
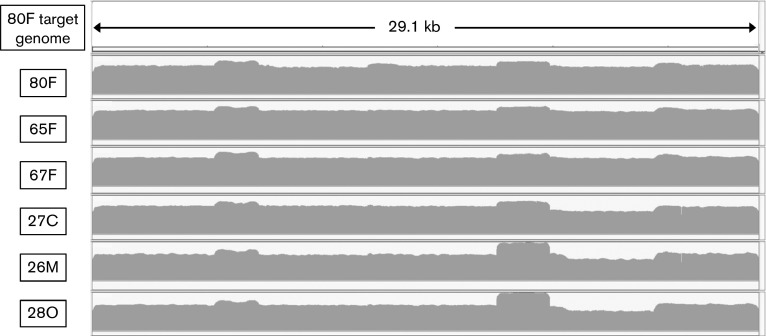
Coverage of sequence reads across the assembled FCoV genomes from faecal and tissue lesion samples. Sequence reads were aligned against the *de novo* assembled 80F target genome for the faeces-derived samples 80F, 65F and 67F and the tissue lesion-derived samples 26M, 27C and 28O.

Using the methods described, we were able to obtain full genome coverage, with a minimum depth of 1000 reads at each base across the coding region (Fig. 3). We expect that, with further optimization, it would be possible to obtain an acceptable level of coverage and depth for more than four complete genomes per single 316v2 chip (see Methods). Similarly, it would also be possible to obtain a very high density of reads for a single genome if, for example, the goal was to investigate the nature of the viral quasispecies in a particular sample. In our opinion, the limiting step in genome sequencing from clinical samples is the production of amplicons, but, once this has been achieved, the downstream processing is relatively straightforward.

Our approach was based on the alignment of sequence reads with a *de novo-*assembled target genome and this is dependent on a relatively high similarity between samples. For example, in the case of the 65F, 67F, 26M and 28O samples, the percentages of reads that aligned with the 80F target genome were 96, 95, 90 and 95 %, respectively. However, only 76.8 % of reads from the 27C sample aligned with the 80F target genome. Thus, for the 27C sample, the *de novo* assembly method had to be used. *De novo* assembly is more time consuming and would not be a good approach if every sample had to be analysed in this manner, as would be the case if they were highly divergent. It should also be noted that, in our analysis, we only compared genome consensus sequences where each position was defined by a single nucleotide. In reality, for any sample, many nucleotide positions are represented by a proportion of different nucleotides. In these cases, we took the majority nucleotide as the consensus nucleotide and did not attempt to delineate different populations in the quasispecies. This means that, when comparing sequences, we were only able to identify mutations throughout the population of genomes and did not conclude that any or all of these mutations were found in a single genomic RNA.

### Phylogenetic analysis

**Fig. 4.**
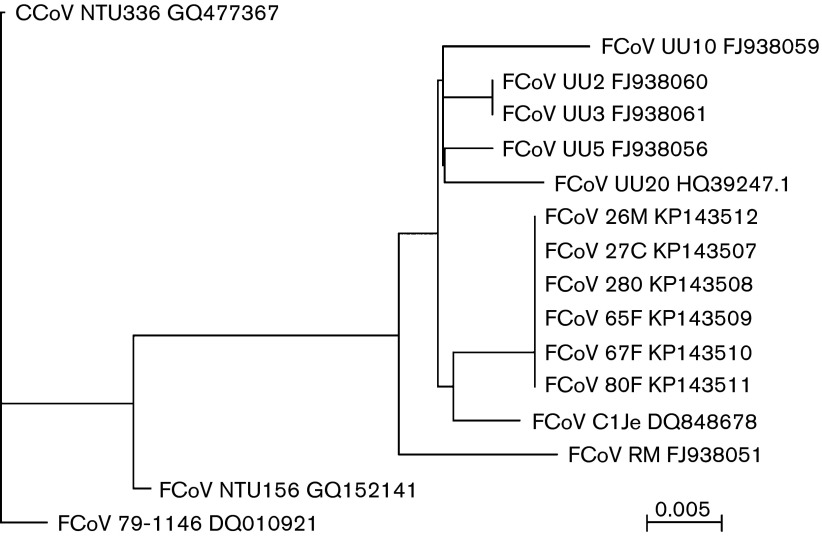
Phylogenetic analysis of the core RdRp domain of nsp12 (aa 4503–4807 in pp1ab, Supplementary Fig. S1 available in the online Supplementary Material) for FCoV strains sequenced in this study and selected FCoV genome sequences. The phylogenetic tree was reconstructed by the neighbour-joining method from an alignment made with clustal w (MacVector). GenBank accession numbers are shown for all sequences. Bootstrap values exceeded 60 % at all nodes. Bar, nucleotide substitutions per site.

Phylogenetic analysis of the six clinical samples described here, based on the conserved RNA-dependent RNA polymerase (RdRp), showed that they comprised a closely related cluster (Fig. 4). As reported by Barker *et al.* (2013), there was no evidence that the samples derived from FIP or non-FIP animals represented genetically diverse co-circulating strains, which provides further support for the ‘internal mutation’ hypothesis. However, it was very difficult to exclude the possibility that at least some of the mutations that may contribute to the development of FIP were present in a minor component of the infecting population, which was subsequently selected during virus replication *in vivo*.

### Comparison of FCoV genome sequences from clinical samples

The genome sequences of the six FCoVs derived from faecal and tissue lesion samples were translated into two polyproteins (pp1a and pp1ab), four structural proteins (S, M, N and E) and five accessory proteins (3s, 3b, 3c, 7a and 7b). We found that amino acid differences were located at 44 positions across an alignment of the six translatomes. At 21 of these positions, the differences fully or partially discriminated between the genomes derived from faecal (i.e. non-FIP) samples and from tissue (i.e. FIP) samples. More specifically, in these 21 positions, one or more of the translatomes from the FIP samples displayed an amino acid that was not found at the corresponding position in the translatomes from any of the non-FIP samples (Table 1). We also identified deletions in the 3c protein ORF of genomes from two of the FIP samples.

**Table 1.** Amino acid substitutions that partially or fully discriminate between the genome sequences derived from non-FIP (faecal) and FIP (tissue lesion) samples The positions of the substitutions are indicated as the position of the relevant mutation/substitution based on alignment of the six FCoV genomes analysed in this study (Supplementary Fig. S1). The amino acid positions in the non-structural replicase proteins (nsps) refer to pp1ab. The consensus nucleotide constituted more than 96 % of the sequence reads at a given position, unless indicated otherwise.

Nt position	Protein	65F	67F	80F	26M	27C	28O	Aa position
1758	nsp2	I	I	I	V	V	I	549
1794	nsp2	G	G	G	G	G	R	561
6553	nsp3	D	D	D	G	G	D	2147
14 727	nsp12	Y	Y	Y	F*	F*	Y	4872
17 883	nsp14	T	T	T	I	I	T	5924
19 277	nsp16	N	N†	N	H	H	N	6389
21 370	S	S	S	S	A	S	S	403
21 377	S	I	I	I	I	I	T	405
22 291	S	F	F	F	F	F	L	710
22 361	S	S	S	S	I	I	S	733
22 528	S	R	R	R	G	G	R	789
22 539	S	R	R	R	R	R	S	792
22 757	S	S	S	S	F	F	S	865
23 302	S	M	M	M	L	L	L	1047
23 486	S	I	I	I	T	T	T‡	1108
23 589	S	K	K	K	N	N	K	1142
24 190	S	P	P	P	P	P	S	1343
24 298	S	E	E	E	Q	Q	E	1379
25 447	3C	T	T	T	T	T	M	165
25 580–25 589§	3C	(−)	(−)	(−)	(+)	(+)	(−)	Deletion
27 228	N	S	S	S	L	L	S	170
28 759	7B	L	L	L	F	L	L||	198

* The consensus nucleotide constituted 83 % (26M) and 55 % (27C) of the sequence reads at this position.

† The consensus nucleotide constituted 60 % of the sequence reads at this position.

‡ The consensus nucleotide constituted 75 % of the sequence reads at this position.

§ (−), 3c protein gene was complete; (+), 3c protein gene had a deletion. The deletions were: 26M and 27C, nt 25584–25593 (10 nt, AGGAGTTTAC).

||The consensus nucleotide constituted 85 % of the sequence reads at this position.

The fully discriminatory differences we identified were located at two positions where a different amino acid was found in all three FIP translatomes compared with all three non-FIP translatomes. The first of these was at nt 23 302 and corresponded to the methionine-to-leucine substitution identified by Chang *et al.* (2012). Thus, our data support the idea that this substitution may be critical with regard to the pathogenesis of FIP. The second fully discriminatory substitution we identified, which was present in all of the FIP samples but none of the non-FIP samples, was at nt 23 486 and resulted in an isoleucine-to-threonine substitution in the heptad repeat region 1 (HR1) of the S2 domain in the FCoV S protein. The possible significance of this substitution is discussed in more detail below.

Apart from the fully discriminatory substitutions described above, Table 1 shows a further 19 positions where one or two of the translatomes from the FIP samples displayed an amino acid that was not found at the corresponding position in the translatomes from non-FIP samples. Without any further information, it is difficult to conclude that any of these substitutions, alone or in combination, may be related to the development of FIP. However, they should not be ignored. For example, the substitutions resulting from mutations at nt 22 528 and 22 539 both lie within the furin cleavage motif that separates the S1 (receptor-binding) and S2 (fusion) domains of the FCoV S protein. Both substitutions (R789G at P4 and R792S at P1, where P4 and P1 designate positions in the canonical furin cleavage motif) would be predicted to alter furin cleavage activity. If this is the case, our results support the conclusions of Licitra *et al.* (2013) who identified the furin cleavage site as a potentially important region in the development of FIP. Alternatively, it could be argued that once the virus has acquired a tropism for the monocyte/macrophage, cleavage at the furin recognition motif may no longer be relevant to virus entry and mutations may accumulate due to a lack of selection pressure. For coronaviruses such as mouse hepatitis virus, cleavage at the canonical furin motif does not seem to be essential, at least for *in vitro* infectivity (Bos *et al.*, 1997), and recent results suggest that activation of the coronavirus S protein fusion activity requires proteolytic cleavage at a different position in the S2 subunit (Millet & Whittaker, 2014; Wicht *et al.*, 2014). Finally, Table 1 shows that two of the three translatomes derived from the FIP samples had a deletion in the 3c protein gene, which was not found in any of the non-FIP samples. In both cases, the deletion of 10 nt led to a translational frameshift that produced a 3c protein truncated 8 aa downstream of the deletion site.

In addition to amino acid substitutions that partially or fully discriminated between the genomes derived from non-FIP and FIP samples, our study also identified a further 23 amino acid substitutions that did not discriminate between non-FIP and FIP genomes. These are listed in Table 2. These substitutions will not be discussed in detail, but it is, perhaps, worth noting that the majority were found either in the nsp3 protein or in the amino-proximal S1 region of the S protein. This suggests that these regions may represent the targets of particularly strong selective pressures. In the case of the S1 region of the S protein, we speculate that this selective pressure is immunological and relates to the production of neutralizing antibodies. The selective pressures that target the nsp3 protein are unknown. For completeness, we also note that we identified a single G-to-T mutation in the 3′ UTR at nt 28 926 of the consensus sequence derived from the 26M sample that was not found in any other sample.

**Table 2.** Amino acid substitutions that do not discriminate between the genome sequences derived from non-FIP (faecal) and FIP (tissue lesion) samples The positions of the substitutions are indicated as the position of the relevant mutation/substitution based upon an alignment of the six FCoV genomes analysed in this study (Supplementary Fig. S1). The amino acid positions in the nsp proteins refer to pp1ab. The consensus nucleotide constituted more than 96% of the sequence reads at a given position, unless indicated otherwise.

Nt position	Protein	65F	67F	80F	26M	27C	28O	Aa position
813	nsp2	V	I	I	I	I	V	234
1794/1795	nsp2	E	G	G	G	G	R	561
2955	nsp3	A	T*	T	T	T	A	948
3082	nsp3	R	K†	K	K	K	R	990
3797	nsp3	Q	Q	H	Q	Q	Q	1228
5218	nsp3	V	A	A	A	A	V	1702
5337	nsp3	L	M	M	M	M	L	1742
6804	nsp3	A	S	A	A	A	A	2231
8939	nsp5	K	K	N	N	N	K	2942
14 564	nsp12	A	A	P	P	P	A	4818
15 185	nsp13	I	I	L	I	I	I	5025
20 509/20 510	S	A	P‡	L	P	P	A	116
20 584	S	D	N	N	N	N	D	141
20 861	S	S	S§	N	S	S	S||	233
20 864	S	R	Q	R	Q	Q	R	234
20 866	S	I	L¶	I	I	I	L	235
21 275	S	R	R#	Q	R	R	R	371
21 467	S	I	T	T	T	T	I	435
22 151	S	R	K**	K	R	R	R	663
22332	S	I	I††	M	I	I	I	723
27273	N	L	Q	Q	Q	Q	L	185
27873	7A	H	Y‡‡	H	Y	Y	H	6

* The consensus nucleotide constituted 71 % of the sequence reads at this position.

† The consensus nucleotide constituted 72 % of the sequence reads at this position.

‡ The consensus nucleotide constituted 71 % of the sequence reads at this position.

§ The consensus nucleotide constituted 68 % of the sequence reads at this position.

||The consensus nucleotide constituted 85 % of the sequence reads at this position.

¶ The consensus nucleotide constituted 71 % of the sequence reads at this position.

# The consensus nucleotide constituted 60 % of the sequence reads at this position.

** The consensus nucleotide constituted 59 % of the sequence reads at this position.

†† The consensus nucleotide constituted 56 % of the sequence reads at this position.

‡‡ The consensus nucleotide constituted 78 % of the sequence reads at this position.

## Discussion

This study demonstrated an approach to the complete genome sequencing of FCoVs derived from clinical material that is achievable in a standard laboratory setting. It was based on the generation of a virus-specific cDNA library using oligonucleotide primer pairs, followed by next-generation sequencing (NGS) on a commercial platform, and downstream genome assembly using free software that will run on a personal computer. This approach was taken after we had failed to determine complete genome sequences of FCoV from clinical samples using a randomly primed cDNA library followed by NGS (Porter, 2014). In the study reported here, complete genome sequencing was achieved for six FCoVs using only seven primer pairs. However, the samples we used were all collected within a few months at a single location, which means that they were less likely to have diverged compared with samples taken at different locations over a longer time period. As the number of complete genome sequences for both serotype 1 and serotype 2 FCoVs increases, it may be possible to design a set of universal primer pairs that will only require minor optimization to successfully sequence any FCoV genome. In our own laboratory, we have shown that the seven primer pairs described here are able to produce amplicons of the expected size in approximately two-thirds of geographically divergent UK faecal samples collected over a 2-year period (unpublished results).

In addition to confirming earlier findings, the most interesting result of this study is undoubtedly the identification of a consistent substitution of isoleucine with threonine at aa 1108 in all FCoVs from FIP lesions compared with the faecal samples from healthy cats. This substitution is located within the heptad HR1 region of the S2 subunit of the FCoV S protein and could be interesting from two points of view. First, we note that this amino acid position has been identified as being located in a major T-helper 1 (Th1) epitope (I-S2-6, IGNITLALGKVSNAITTTSD) in a type 1 Japanese FCoV (KU-2) that was associated with FIP (Satoh *et al.*, 2011). Obviously, further research will be required to ascertain whether there is a Th1 epitope spanning this amino acid sequence in non-FIP-associated FCoVs, and to determine the quantitative or qualitative effect that may result from the isoleucine-to-threonine substitution. However, de Groot-Mijnes *et al.* (2005) have already drawn attention to the relationship between T-cell depletion and the enhanced virus replication in FIP cases, although the mechanisms of T-cell depletion are not yet clear. We suggest that this is an area of FIP research that merits further study. For example, it would be interesting to compare IFN-γ production by PBMCs taken from cats with FIP or healthy FCoV-infected cats, and exposed separately to relevant HR1 peptides, the sequences of which are derived from FIP- and non-FIP-associated FCoVs.

Secondly, a quite different interpretation of the HR1 isoleucine-to-threonine substitution is that it may be related to the fusogenic activity of the FCoV S protein. This is because the substitution also lies within a stretch of 15 aa [NAITT(I/T)SDGFNTMAS] that are found only in alphacoronaviruses and are part of the heptad repeat structure that characterizes the HR1 region. Indeed, the isoleucine/threonine position constitutes a residue predicted to be located on the hydrophobic interface of the coiled-coil structure. Substitution of a hydrophobic residue with a polar, uncharged residue may, at least theoretically, significantly influence the intercalation of HR1 and HR2 regions, which is a necessary event during membrane fusion. It is also worth noting that a very recent study by Bank-Wolf *et al.* (2014) identified a position two residues downstream of the isoleucine-to-threonine substitution where an aspartate residue was found in all examined non-FIP-associated FCoVs (5/5) but was replaced by a tyrosine in a significant proportion (5/9) of the FIP-associated FCoVs. Neither the isoleucine-to-threonine nor aspartate-to-tyrosine substitutions consistently discriminated between FIP and non-FIP FCoVs in the wider alignment of 29 type 1 FCoV S protein amino acid sequences that we examined (data not shown) but, again, we think they may represent substitutions that are functionally related and could be relevant to the development of FIP.

The comparative sequence approach taken by ourselves and others has identified a number of potentially interesting mutations in the coding sequences of non-FIP- and FIP-associated FCoVs. In the future, this approach could be extended, i.e. a larger collection of well-defined clinical samples should be analysed, and it can be refined. For example, to distinguish mutations that may relate to the tropism of FCoVs from those that may relate to virulence, we suggest it would be important to obtain sequence data from a virus population that infects monocytes but is not able to replicate at a high level. Clearly, obtaining appropriate clinical samples (e.g. blood monocytes from clinically healthy, FCoV-infected cats) would not be easy, but it would be very illuminating. The idea that a virus has to undergo sequential mutation *in vivo* in order to cause a specific disease is not unique to FIP (see, for example, the review on measles virus pathogenesis by de Vries *et al.*, 2012), but we suggest it deserves closer attention in a number of veterinary and human diseases.

Nevertheless, this sequencing approach is ultimately limited. As has been stated before, compelling evidence that any specific mutation in the FCoV genome is important for the development of FIP will require the use of well-defined and characterized viruses produced by reverse genetics and a valid experimental model of FIP. With respect to reverse genetics, there are a number of robust reverse genetics systems available for coronaviruses in general, and for particular strains of FCoV (namely the type 2 FCoV strain 79-1146 and the cell-culture-adapted type1 FCoV strain Black) (Thiel *et al.*, 2014). The pressing need, however, is for a robust reverse genetics system that can be applied to field strains of type 1 FCoV. In our opinion, the bottleneck is not the molecular manipulation of the FCoV genome but rather the ability to propagate type 1 FCoVs in cell culture without extensive adaptation. Although there has been recent progress in the development of enterocyte cell lines that propagate type 1 FCoVs (Desmarets *et al.*, 2013), we believe that a more robust cell-culture system that allows the propagation of high virus titres and the rescue of both mutated and non-mutated virus will be needed. To achieve this, identification of both the cellular receptor and attachment factors specific to type 1 FCoVs and the transduction of well-established, continuous, feline cell lines that can easily be maintained will be essential.

The second required element, a valid experimental model of FIP, is also more challenging than it may at first appear. For example, many of the commonly used animal models of FIP often involve intraperitoneal inoculation. If the natural course of FCoV infection involves sequential replication in the gut, low-level replication in blood monocytes and high-level replication in monocytes and macrophages, and each transition is associated with the selection of specific mutants, then this has to be reproduced in any valid experimental model. In a recent report, Tekes *et al.* (2012) showed that intraperitoneal infection of cats with a recombinant form of the FCoV 79-1146 strain robustly induced FIP. Strikingly, the virus reisolated from these cats demonstrated that there had been strong selection for a virus that reverted to encode an intact 3c protein. This is, in our view, good evidence that FIP results from an infection that involves initial replication in the gut.

In summary, our results contribute to a better understanding of FCoV genomic mutations that may or may not be used as markers of the virus phenotype. It is also clear from the results that the relationship between the viral genotype and the development of FIP is complex. The further analysis of complete FCoV genomes in defined clinical samples, a robust reverse genetics system that can be applied to field strains of serotype 1 FCoV and the development of valid experimental models of FIP will all be needed to throw further light on this relationship.

## Methods

### 

#### Clinical samples and RNA extraction.

The samples selected for this study were faecal samples from three healthy kittens and post-mortem tissue lesion samples from three kittens with FIP. These samples were all obtained from a previously reported epizootic outbreak at a single-site UK feline rescue centre (Barker *et al.*, 2013). The three tissue lesion samples, designated here as 26M (mesentery), 27C (colonic lymph node) and 28O (omentum), were from cats F/FIP, Z/FIP and J/FIP in a previous study (Barker *et al.*, 2013) and had been collected within 2 h of death, placed in RNAlater (Life Technologies) for 24–48 h at 4 °C and then, after discarding the RNAlater, stored at −80 °C. The diagnosis of FIP was confirmed by post-mortem examination including histopathology and immunohistochemistry for the demonstration of FCoV antigen in lesions (Kipar *et al.*, 1998). The faecal samples (65F, 67F and 80F, previously named #65, #67 and #80) were collected from the healthy cats within 1 month of euthanasia of the cats with FIP (Barker *et al.*, 2013). Samples 80F and 27C were from cats that were littermates and were housed within the same pen. All three cats that provided faecal samples remained alive and without any clinical signs that could be suggestive of FIP for over 1 year post-sampling. Faecal samples were stored at −80 °C immediately after collection.

Total RNA was extracted and purified from 20 mg tissue with a NucleoSpin RNA kit (Macherey-Nagel) based on a previously described method (Dye & Siddell, 2007; Dye *et al.*, 2008). Briefly, 20 mg each tissue sample was disrupted in a 2 ml tube by adding 500 µl lysis buffer containing 1 % β-mercaptoethanol (v/v) and a 5 mm stainless steel ball bearing. The sample was homogenized using a TissueLyser II (Qiagen) at 30 Hz for 2 min and 470 µl lysate was added to a filter column and centrifuged for 30 s at 10 000 ***g***. A 350 µl aliquot of the filtrate was added to 250 µl ethanol and run through a binding column to which DNase I was added to remove genomic DNA. Following multiple washes, the RNA was eluted into 50 µl nuclease-free water. The NucleoSpin RNA kit was also used to extract RNA from faecal samples using a method based on that described by Dye *et al.* (2008). A faecal suspension was produced by vortexing 0.5 g faeces and 4.5 ml PBS five times for 30 s each. Subsequently, 100 µl of this suspension was centrifuged for 2 min at 10 000 ***g***, and the supernatant removed and added to 350 µl lysis buffer containing 1 % β-mercaptoethanol (v/v). The protocol described above (from the filter column) was then followed.

#### Histology and immunohistochemistry.

Formalin-fixed tissue samples (26M, 27C and 28O) were routinely paraffin wax embedded and examined histologically to confirm the presence of typical FIP lesions. The immunohistochemistry served to demonstrate FCoV antigen within the lesions, as described previously (Kipar *et al.*, 1998).

#### Quantitative RT-PCR (qRT-PCR) and virus-specific oligonucleotide primer design.

FCoV RNA was amplified from faecal and tissue samples using qRT-PCR as described previously (Dye *et al.*, 2008; Porter *et al.*, 2014). Oligonucleotide primer pairs (Table 3) were designed to produce a total of seven RT-PCR products (amplicons) spanning the entire coding region of the FCoV genome using the MacVector Primer3 software package. Initially, the primers were designed based on the genome sequence of FCoV C1Je, a serotype 1 FCoV (Dye & Siddell, 2007). The primers were then compared with an alignment of 29 serotype 1 FCoV genome sequences (clustal w; sequences available upon request) and optimized to allow for sequence variation and compatibility of the primer pairs. All primers were synthesized by Eurofins MWG Operon.

**Table 3.** Sequences of oligonucleotide primers used in this study

Name	Amplicon	Sequence (5′→3′)	Position of 5′ nucleotide in C1Je*	Size (nt)
F169	1	TAGGAACGGGGTTGAGAG	169	18
R6507	1	GTGCGAGAACRGCCTTAA	6 473	18
F5562	2	GTTTGAAYTCACGTGGYCATT	5 511	21
R7490	2	GARGTCTTCATCWGAACCCAC	7 459	21
F6943	3	GCTAGTGTTAGAAATGTCTGTGTT	6 923	24
R12466	3	AAAAGCCCTACTAACGTGGTC	12 435	21
F12224	4	CATCCTGCAATTGAYGGATTG	12 173	21
R18105	4	TCCGGGTACATGTCTACGTTA	18 074	21
F17830	5	GATTGGTCCATTGTGTACCC	17 782	20
R20131b	5	AAARCCTTCCGATGACGAGGT	20 100	21
F19786b	6	GTATTAAGRAGATGGTTGCCA	19 735	21
R26007	6	ATAACCGCATGAGAAAAGGCT	25 813	21
F24798	7	TAAAATGGCCKTGGTATGTGT	24 601	21
R29508	7	TAGCTCTTCCATTGTTGGCTC	29187	21

* The positions of the oligonucleotides are given relative to the genome of FCoV C1Je (GenBank accession no. DQ848678).

#### One-step RT-PCR.

FCoV-specific primers were used to reverse transcribe and amplify the viral RNA contained in 2 µl extracted total RNA using a SuperScript III One-Step RT-PCR System with Platinum *Taq* High Fidelity (Life Technologies) as described by the manufacturer. Briefly, a 50 µl reaction was set up on ice containing 2 µl RNA, 1 µl 10 µM forward and 1 µl 10 µM reverse primer, 25 µl 2× reaction mix (as supplied by the manufacturer), 2 µl SuperScript III RT/Platinum High Fidelity enzyme mix and water to a final volume of 50 µl. The reaction was incubated at 50 °C for 50 min to allow cDNA synthesis, and then raised to 94 °C for 2 min, followed by 41 cycles of 94 °C for 15 s, 50–66 °C (depending on the primer set) for 30 s and 68 °C for 1 min (kb of product size)^−1^. The annealing temperature for individual reactions was determined by the melting temperature of the primers used. The reaction underwent a final extension phase at 68 °C for 7 min and was held at 4 °C. For each amplicon, 5 µl PCR product was separated on a 1 % agarose/TBE gel to confirm the PCR product size and to estimate the amount of DNA by comparison with standards. The PCR products were then pooled in approximately equimolar amounts and purified using Agencourt AmPure XP beads (Agencourt AMPure XP PCR Purification; Beckman Coulter), following the manufacturer’s protocol, and eluted in nuclease-free water.

#### NGS.

Purified, pooled amplicons were sequenced at the University of Bristol Genomics Facility using the Ion Torrent platform (PGM with a 316v2 chip). A targeted, virus-specific cDNA single-end read library was produced. Briefly, DNA was fragmented using an Ion Xpress Plus Fragment Library kit, ligated to Ion-compatible barcoded adaptors and size-selected for a target read length of 150–200 bases. The library was then amplified and purified using an Ion Plus Fragment Library kit and an Agencourt AMPure XP kit. The barcoded libraries were quantified and pooled in equimolar amounts using Bioanalyser quantification. Templates were prepared from the barcoded, pooled libraries using an Ion OneTouch 2 System. Routinely, four genomes were sequenced on a single 316v2 chip.

#### Bioinformatics.

**Fig. 5.**
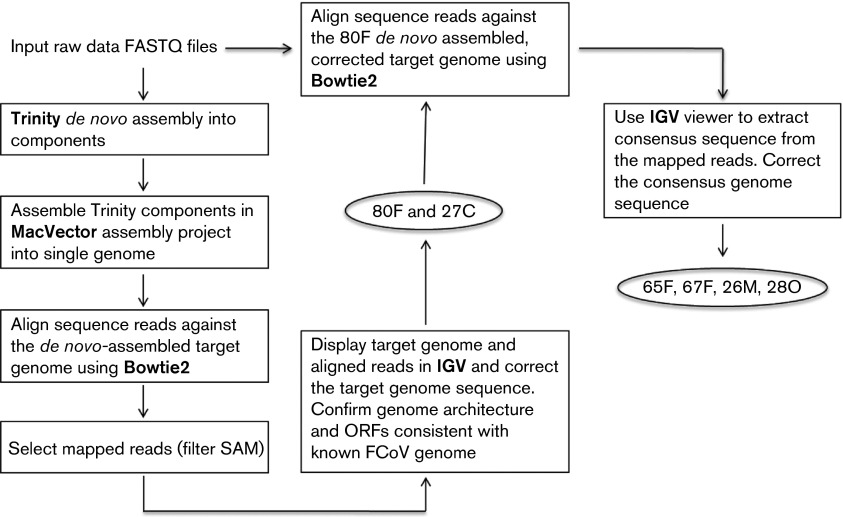
Sequence assembly workflow for FCoV genomes from faecal samples 65F, 67F, 80F and tissue lesion samples 26M, 27C, 28O.

Sequence data were analysed using bioinformatics tools including both *de novo* assembly (Trinity, http://trinityrnaseq.github.io/) and genome alignment (Bowtie2, http://bowtie-bio.sourceforge.net/bowtie2/index.shtml) methods. Briefly, for samples 80F and 27C, a *de novo* consensus sequence was produced from the FASTQ reads using the Trinity assembled components and the MacVector assembly project tool (Grabherr *et al.*, 2011). In order to identify and correct possible errors in this assembly, the same fastq sequence files were then aligned with the assembled consensus sequence using Bowtie2. The alignments were visualized using the Integrative Genomics Viewer (IGV) and the consensus sequence was manually corrected on the basis of the sequence reads. Subsequently, the fastq sequence reads for four samples (65F, 67F, 26M and 28O) were aligned with the corrected 80F consensus sequence using Bowtie2. Again, IGV was used to confirm each consensus sequence with regard to the relevant sequence reads. All of the assembled genome sequences were examined and confirmed to have the expected FCoV genome architecture and predicted ORFs. This workflow is illustrated in Fig. 5. For selected viral genes, the encoded protein sequences were derived and phylogenetic reconstruction was done using a neighbour-joining algorithm based on an alignment generated by clustal w (MacVector).
